# Fetal-onset malignant rhabdoid tumor: a case report

**DOI:** 10.1186/s13256-022-03503-7

**Published:** 2022-07-20

**Authors:** Ryota Kobayashi, Wakako Sumiya, Toshiyuki Imanishi, Chika Kanno, Masayuki Kanno, Jun Unemoto, Ken Kawabata, Masami Kanno, Masaki Shimizu

**Affiliations:** 1 Department of Pediatrics, Jikei University School of Medicine, 3-25-8, Nishi-Shimbashi, Minato-ku, Tokyo, 105-8461 Japan; 2 Department of Neonatology, Saitama Children’s Medical Center, 1-2, Shintoshin, Chuo-ku, Saitama-shi, Saitama 330-8777 Japan

**Keywords:** Fetal diagnosis, Ex-utero intrapartum treatment, Malignant rhabdoid tumor, Fetal onset

## Abstract

**Background:**

A fetal-onset cervical mass may cause postnatal airway obstruction, and ex utero intrapartum treatment (EXIT) to secure the airway while maintaining fetal-placental circulation may be life-saving. Malignant rhabdoid tumors (MRT) are highly aggressive tumors, and when they develop in utero, the prognosis is even worse, with almost no reports of survival beyond the neonatal period. Herein, we report a case of a primary cervical MRT and describe our treatment using EXIT for securing the airway, wherein the infant’s life was saved.

**Case presentation:**

A 40-year-old Japanese woman with no relevant medical or surgical history was diagnosed with a fetal left cervical mass and polyhydramnios during the third trimester. Fetal magnetic resonance imaging indicated the possibility of postnatal airway obstruction, and delivery using EXIT was planned. The infant was delivered by a planned cesarean section at 39 weeks and 5 days gestation, and tracheostomy was performed using EXIT. Postnatal contrast-enhanced computed tomography revealed suspected metastatic lesions in the subcutaneous tissue, lungs, and thymus, in addition to the mass in the left cervical region. MRT was diagnosed by biopsy of a subcutaneous mass in the left thigh, and chemotherapy with vincristine, doxorubicin, cyclophosphamide, ifosfamide, and etoposide was initiated. The tumors regressed, and the infant was successfully weaned from artificial ventilation. After discharge from the hospital, she had a recurrent cervical mass and intracranial metastasis, and radiotherapy was initiated.

**Conclusions:**

In our case, fetal diagnosis enabled advance planning of delivery using EXIT, thus saving the infant’s life. The use of chemotherapy for MRT, which has a poor prognosis, allowed tumor regression and enabled the infant to survive beyond the neonatal period.

## Background

The widespread use of fetal diagnosis is enabling therapeutic interventions not only immediately postnatally but also prenatally. Fetal-onset cervical masses may cause postnatal airway obstruction, and ex utero intrapartum treatment (EXIT) that maintains the fetal-placental circulation and secures the airway can be life-saving for infants [1].

Reaching a definitive diagnosis of the mass immediately after delivery and selecting appropriate treatment methods are also important. Malignant rhabdoid tumors (MRT), the malignancy present in our patient, are highly aggressive tumors with strong resistance to treatment and a high mortality rate [2], and the correct choice of treatment will improve the quality of life (QOL) of both the infant and their family.

Fetal-onset MRT has an even worse prognosis. The only reported case of survival beyond the neonatal period was that of a single patient with an atypical teratoid rhabdoid tumor (AT/RT) [3]; however, to the best of our knowledge, no such cases of extracranial MRTs have been reported.

Herein, we report a case of an infant with airway obstruction by a fetal cervical mass that was diagnosed prenatally, wherein delivery using EXIT was planned, and describe the successful treatment that was life-saving. The appropriate therapeutic intervention resulted in contraction of the mass, enabling the infant to survive beyond the neonatal period, despite the poor prognosis of MRT.

## Case presentation

**Fig. 1:**
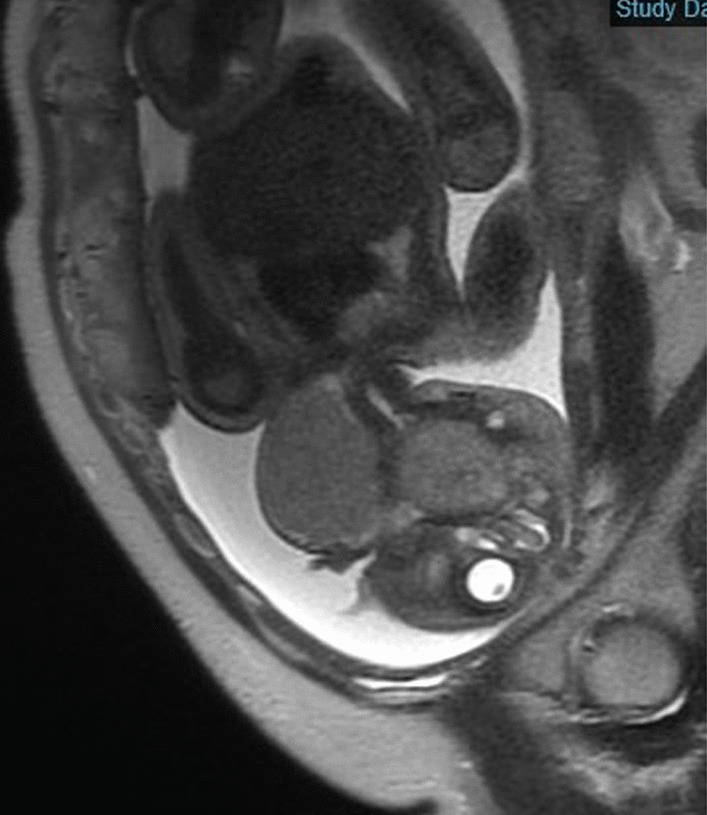
Nodule formation. Two nodules measuring 41 × 44 mm and 53 × 47 mm formed a continuous mass from the base of the tongue to the left buccal region

A 40-year-old Japanese primigravida nullipara underwent fetal ultrasound at 39 weeks and 3 days gestation that revealed a left cervical mass and polyhydramnios. This had not been noted in fetal screening during the first or second trimesters and was only identified in the third trimester. Fetal magnetic resonance imaging (MRI) scanning was performed the same day (Fig. 1), and the mass was found to consist of a massive solid component located at the base of the tongue adjacent to the airway. Given that postnatal airway obstruction or stenosis was a concern, the patient was transferred to a high-level children’s general hospital, which also included a department of obstetrics, to perform EXIT.

The infant was delivered by a planned cesarean section, and a simple tracheostomy was conducted using EXIT. The infant was a girl born at 39 weeks and 5 days gestation, with Apgar scores of 2, 3, and 4 (1, 5, 10 minutes, respectively). After the airway had been secured using EXIT, she was admitted to the neonatal intensive care unit, sedated, and placed on artificial ventilation. On physical examination, her birth weight was 2870 g [– 0.3 standard deviation (SD)], head circumference was 33.5 cm (+ 0.1 SD), and body length was 51 cm (+ 1.0 SD). In addition to the left cervical mass and the mass at the base of the tongue diagnosed on fetal MRI, multiple subcutaneous lesions were also noted on the limbs and trunk. Blood tests showed that lactate dehydrogenase level was elevated at 2470 IU/L, alpha fetoprotein level was 42,580 ng/mL, human chorionic gonadotropin level was 19.3 mIU/L, and vanillylmandelic acid and homovanillic acid test results were negative.

Cervical and surface ultrasound scanning conducted on postnatal day 0 revealed that the left cervical mass was an internally heterogeneous solid mass measuring 40 × 32 × 49 mm, with an abundant blood flow signal. A hyperechoic region was evident and considered to represent calcification. The multiple subcutaneous lesions included some with abundant blood flow and some with little blood flow containing hypoechoic regions.

**Fig. 2:**
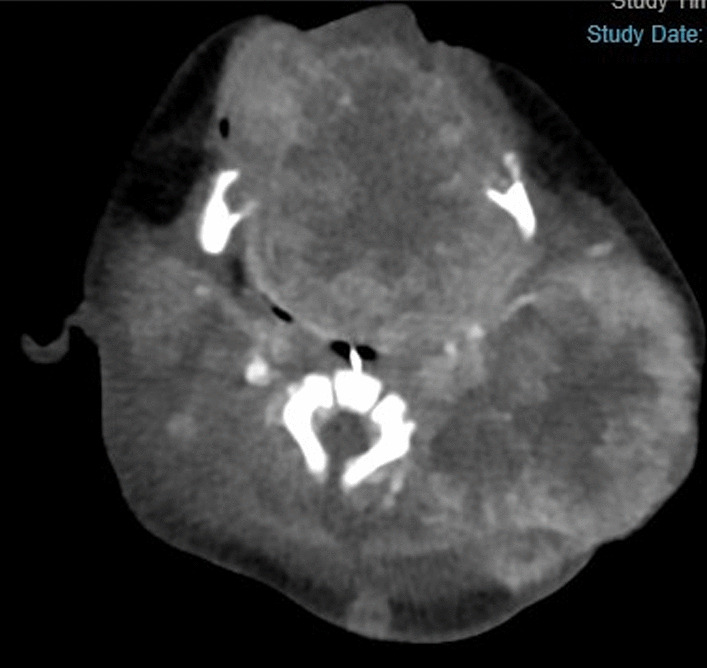
Contrast enhancement is evident at the margins of the mass but is lacking at its center, which was necrotic

**Fig. 3:**
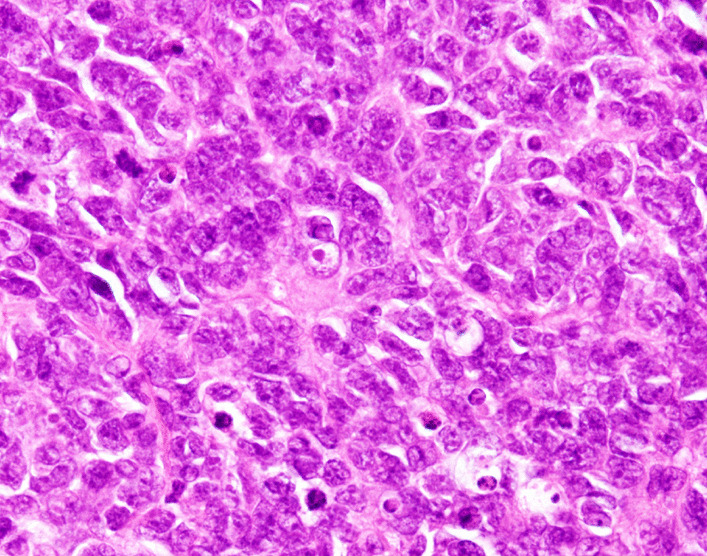
Hematoxylin and eosin staining of the subcutaneous mass in the left thigh

**Fig. 4:**
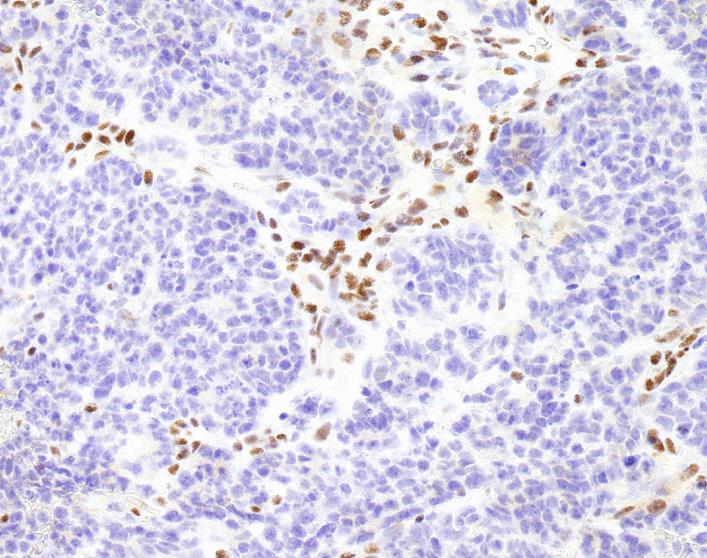
SNF-related, matrix-associated, actin-dependent regulator of chromatin subfamily B member 1/integrase interactor 1 (SMARCB1/INI1) intranuclear expression is lost

Contrast-enhanced computed tomography conducted on postnatal day 5 (Fig. 2) revealed that the interior of the mass was severely necrotic. Masses were also present in the lower lobe of the right lung and the thymus and were considered to be metastases. There was no invasion of the cerebral parenchyma. A tumor biopsy was performed from a subcutaneous mass in the inner left thigh on postnatal Day 1. Hematoxylin and eosin staining (Fig. 3) revealed a dense proliferation of undifferentiated tumor cells with round nuclei. Sites of hemorrhage and necrosis were present, and numerous mitotic figures and karyorrhexis were apparent. On immunostaining, the tumor cells were negative for CD3, CD20, CD45, CD99, desmin, myogenin, Nkx2.2, PHOX2B, tyrosine hydroxylase. Intranuclear expression of SMARCB1(INI1) was also absent (Fig. 4). From these results, MRT was diagnosed.

**Fig. 5:**
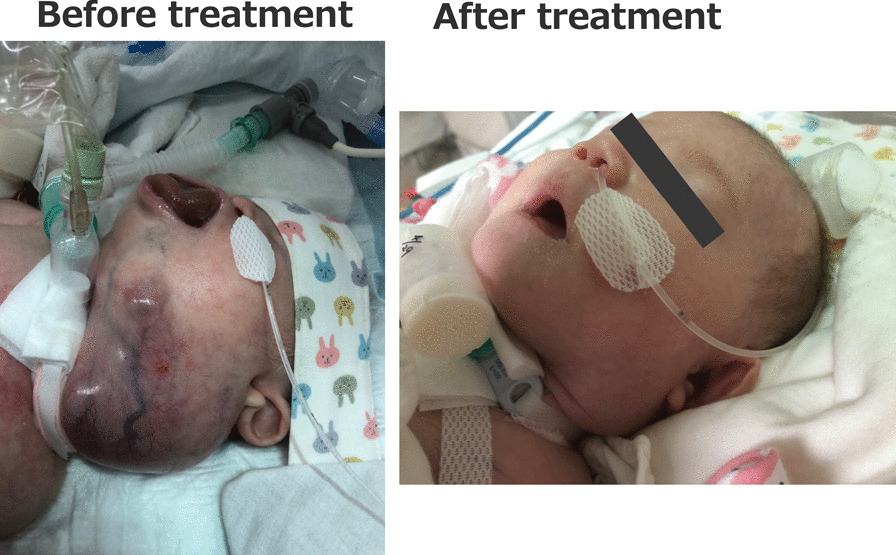
Before and after treatment

Chemotherapy with vincristine, doxorubicin, cyclophosphamide, ifosfamide, and etoposide was initiated for the MRT on postnatal day 12. Mild myelosuppression occurred, but this was not sufficiently severe to warrant treatment discontinuation. Renal impairment and tumor lysis syndrome did not occur as chemotherapy side effects, and it was possible to continue treatment. At 2 months of age, the left cervical mass had clearly regressed (Fig. 5), and the infant was successfully weaned from artificial ventilation. She was discharged home at 6 months of age. Neonatal follow-up was performed, and at 9 months of age the patient had a recurrent cervical mass and intracranial metastasis, and radiotherapy was initiated.

## Discussion and Conclusions

This study presents the findings of our case, wherein a fetal patient was diagnosed in utero with a cervical mass with the potential to cause postnatal fatal airway obstruction, and the infant’s life was saved by performing a tracheostomy using EXIT. Postnatal immunohistological testing led to an immediate diagnosis of fetal-onset MRT, and chemotherapy caused the tumor to regress, thus enabling the discharge of the infant and avoiding neonatal death.

In our case of a fetal-onset cervical mass, we successfully secured the airway by EXIT. The indications for EXIT are (1) expanding tumors of the head and neck or the oral region to congenital airway stenosis and (2) congenital pulmonary airway malformation [4]. Moreover, reports of its use to secure the airway have been increasing in recent years, and its safety and efficacy have become well established [5, 6]. However, EXIT is not indicated in all cases of tumors of the head and neck, and the decision must be made based on the nature and location of the tumor. The presence of polyhydramnios, which reflects impaired passage through the esophagus as a result of pressure from the mass, is an important sign [7]. In our case, because fetal MRI revealed a solid mass and polyhydramnios, postnatal airway obstruction was anticipated, and delivery by EXIT was planned. This case re-affirms the importance of fetal diagnosis, which enabled us to conduct a planned delivery and resuscitate the infant using EXIT to secure the airway.

In our study, immunohistological investigations were useful for the definitive diagnosis of MRT. Congenital cervical masses include branchial cleft cysts, vascular malformations, teratomas, thyroid goiters, and neuroblastomas [8]. When proceeding with the differential diagnosis, the onset timing, anatomical location, internal structure, and properties were taken into consideration. In our case, necrotic tumorous lesions and multiple metastatic lesions were evident, and malignancies, including neuroblastoma and MRT, were therefore considered. MRTs have characteristic pathological findings, with rhabdoid cells having eosinophilic cytoplasm containing inclusion bodies and with eccentric nuclei [9]. Mutations in the tumor-suppressor gene SMARCB1(INI1) in the 22q region are characteristic, and a negative result for SMARCB1 on immunohistological staining is a specific finding [10]. An accurate diagnosis is essential for the treatment of malignant tumors, and immunohistological staining is an important technique when proceeding with diagnosis and treatment.

In our case of fetal-onset MRT, the prognosis was anticipated to be extremely poor [3]. However, neonatal death was avoided, and the patient was discharged home. MRTs are highly aggressive and generally fatal. Although standardized epidemiological data are lacking in the literature, a study from the UK has reported a 1-year survival rate of 31% in 106 patients with extracranial MRT [11]. Reports from the European Paediatric Soft tissue sarcoma Study Group and a single Chinese center also provided similar findings [12, 13]. Reportedly, young age at onset is a poor prognostic factor [13]. According to a systematic review, the neonatal survival rate of 72 patients with MRT that appeared in utero or during the neonatal period was 9.7% [3]. In previous studies, metastatic lesions were already present at birth in all 14 patients diagnosed with MRT in utero, wherein 13 patients died within a few days of birth, and although the remaining one patient with an AT/RT survived for 13 months, severe mental retardation and residual brain tumor lesions were evident [3, 14, 15]. In the case reported by Tergestina* et al.* in which the fetus was also delivered by EXIT, the infant survived birth but died of tumor lysis syndrome after 4 days [15]. This case suggests that MRT is even more fatal when it arises in utero, as noted in our patient.

In our case of fetal-onset MRT, metastases were already present at birth. Based on findings from previous reports [13, 14], the prognosis was expected to be poor. After a definitive diagnosis of MRT, we immediately initiated chemotherapy, with the objectives of improving the infant’s QOL and giving her more time with her family. The infant responded well to treatment, the tumor shrank successfully, and she was discharged home at 6 months of age. Compared with previous reports, our study indicates that long-term survival is possible and that continued follow-up is desirable.

In conclusion, we planned delivery by EXIT in advance as a result of the fetal diagnosis, thereby avoiding postnatal airway obstruction. We initiated chemotherapy for fetal-onset MRT, which has an extremely poor prognosis, and succeeded in shrinking the tumor and avoiding neonatal death, while improving the infant’s QOL and giving her time with her family.

## Data Availability

Not applicable.

## Abbreviations

AT/RT: Atypical teratoid rhabdoid tumor
EXIT: Ex-utero intrapartum treatment
MRI: Magnetic resonance imaging
MRT: Malignant rhabdoid tumor
QOL: Quality of life
